# Anterior Aesthetic Rehabilitation for Midline Diastema Closure With Veneers: A Case Report

**DOI:** 10.7759/cureus.49704

**Published:** 2023-11-30

**Authors:** Prasanna R Sonar, Aarati S Panchbhai, Sanket Vaidya

**Affiliations:** 1 Oral Medicine and Radiology, Sharad Pawar Dental College and Hospital, Datta Meghe Institute of Higher Education and Research, Wardha, IND; 2 Dentistry, Sharad Pawar Dental College and Hospital, Datta Meghe Institute of Higher Education and Research, Wardha, IND; 3 Conservative Dentistry, Shri Vasantrao Naik Government Medical College, Yavatmal, IND

**Keywords:** aesthetics, porcelain laminate veneers (plvs), veneers, porcelain, diastema

## Abstract

The value of having excellent teeth has increased in today's culture, even from a merely cosmetic perspective. There are several ways to address issues that arise in the area of high aesthetic sensitivity. Every modality has its benefits and drawbacks. One of the most aesthetically pleasing restorations is porcelain laminate veneers (PLVs). It has been demonstrated that the use of PLVs to address functional and/or aesthetic issues is a viable treatment option, particularly in the anterior aesthetic zone. As long as suitable procedures and guidelines are followed, these restorations are conservative and long-lasting. A patient with an anterior diastema is the main focus of this case study. This case report is essential for expanding dental knowledge, training dental practitioners, proving the effectiveness of treatments, and directing treatment choices for instances that are comparable to it. PLVs were used on the patient to close the diastema in the anterior maxillary arch and achieve a good cosmetic outcome.

## Introduction

A person's confidence is a crucial component of their personality, and a confident smile completes the image. Simply from a cosmetic point of view, tooth appearance has become more and more significant in today's society. People are now beginning to value having a beautiful, healthy smile more. The field of cosmetic dentistry has advanced, providing dental professionals with new avenues for conservative and aesthetically pleasing restoration modalities.

Maxillary midline diastema is a common aesthetic complaint from patients. It is a space bigger than 0.5 mm between the proximal surfaces of the two central incisors [1]. During primary and mixed dentition, the spacing might be a typical growth characteristic. When the permanent maxillary canines emerge, the space is usually closed. However, the diastema does not close on its own for certain people. It can be among the worst things about how one feels about their dental appearance. Functional goals are secondary to aesthetic and psychological goals when it comes to treatment [2].

Currently, several minimally invasive treatments are available to restore and maximize the aesthetics of anterior teeth [3]. The most widely used least invasive methods for closing anterior space and restoring the smile's natural appearance are still indirect ceramic veneers and direct composite restorations. Numerous therapy forms for diastema closure have been created in response to patients' aesthetic desires. The practitioner should not, however, believe that every diastema requires rectification. To guarantee an acceptable aesthetic outcome, the patient's wants, requirements, and expectations must be considered during the treatment planning process. In clinical practice, treating diastema closure aesthetically can be difficult. Porcelain laminate veneers (PLVs), which are thin ceramic shells that are attached to the facial surface of teeth using modern bonding agents and light-cure resin cements, are one of the recommended treatment choices for these issues [4].

Comparing laminate veneers to ceramic crowns, another treatment option, involves less tooth reduction, partial covering, and yet a beautiful result [5-8]. Tooth preparations are often not widely regarded because of the move toward conservative measures for both patients and dentists. As a result, composite laminate veneers made using direct technique gained popularity and were acknowledged by certain dentists as an alternate form of therapy. Nonetheless, there are still several drawbacks to this method, including the inability to fabricate using indirect technology, polymerization shrinkage of the restorative materials, lack of color modifications, polishing, and yellowing of composite materials. This article describes a case with midline diastema and spacing in the maxillary anterior region that was treated conservatively with PLVs to achieve the intended cosmetic outcomes.

## Case presentation

**Figure 1:**
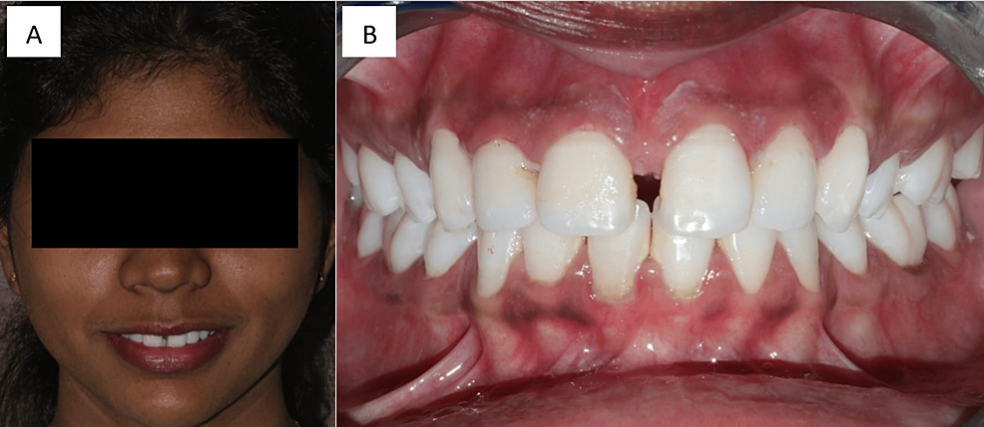
A. Frontal view of the patient. B. Intraoral photograph of the patient. Image Credit: Sanket Vaidya

A 28-year-old woman reported to a dental hospital to have her midline diastema, the space between her maxillary central teeth, corrected (11 and 21). Figure 1 shows the frontal view and intraoral photograph of the patient. Because she was self-conscious about the way her teeth looked, the patient refrained from smiling. She had previously been referred to the orthodontic department, but she refused to have orthodontic intervention because she wanted results immediately.

A dental examination indicated that the maxillary anterior teeth had a diastema. Also, there was mild spacing in the maxillary lateral and canine region bilaterally. There were no cavities in the teeth. The patient has a 2 mm overbite and overjet in a class 1 molar-canine relationship. Diagnostic models were created, impressions were obtained, and they were mounted on a semi-adjustable articulator. The size and form of the restorations were determined by examining the model using computer-aided design and manufacturing (CAD/CAM). Before treatment, a diagnostic wax-up was performed to ensure that the teeth were properly placed and aligned so that the midline diastema was covered and appropriate gingival zenith achieved for each tooth. As a conservative alternative to complete coverage for enhancing the appearance of anterior teeth, laminate veneer preparations for six neighboring teeth were scheduled. It was also chosen since it required very little preparation: only enamel and just enough to give the new surface the proper contour.

**Figure 2:**
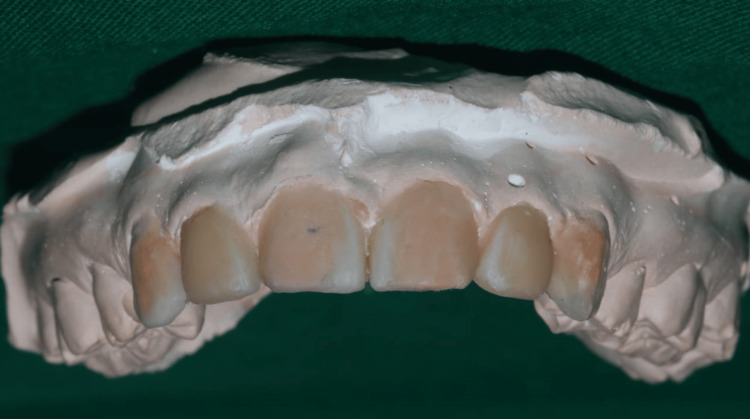
Diagnostic wax-up. Image Credit: Sanket Vaidya

**Figure 3:**
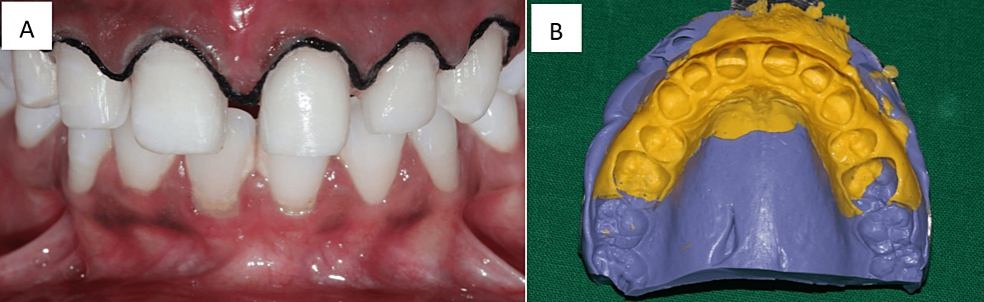
A. Teeth preparation followed by gingival retraction. B. Recorded impression with polyvinylsiloxane impression material in putty and light body consistency. Image Credit: Sanket Vaidya

**Figure 4:**
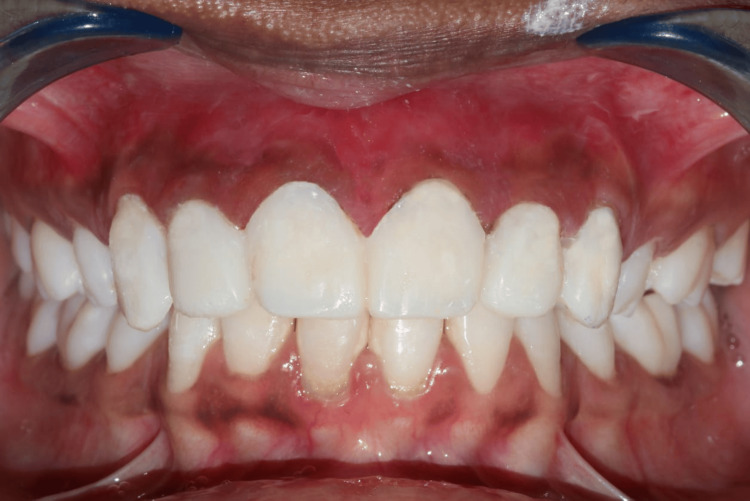
Temporization done with prepared teeth. Image Credit: Sanket Vaidya

Using a diagnostic wax-up, the models were examined to determine the size and form of the restorations as shown in Figure 2. The patient was given the choice of PLV as a treatment for a long time. The patient opted to have the maxillary diastema corrected. Using the VITAPAN classical shade guide (VITA Zahnfabrik, Bad Säckingen, Germany), the shade was chosen before starting the teeth preparation process. After that, the maxillary central incisors were ready for PLVs. Using a tapered diamond bur with a diameter of 1 mm and a depth-cutting diamond bur, the tooth preparation was done in enamel at a depth of 0.5 mm. The cervical area retained a 0.25 mm chamfer. The gingival margin was maintained as the level for the chamfer finish lines. To prevent the margin from being visible and a black triangle from appearing, the proximal preparation was extended beyond the contact region. A 1.5 mm incisal edge reduction was made. The gingival retraction was carried out using size 00 for 6 number of gingival cord. A full-arch impression is made using a one-step technique with polyvinylsiloxane impression material. While the heavy-bodied impression was being poured into the plastic tray and placed inside the oral cavity, the light-bodied impression material was being equally applied to the teeth and softly blown over the preparation. After the impression material had been allowed to set, it was taken out. Figure 3 illustrates teeth preparation followed by gingival retraction and the recorded impression with polyvinylsiloxane impression material in putty and light body consistency. The putty index was used from the mockup to create temporary veneers with Luxatemp material (DMG America, Ridgefield Park, New Jersey, United States). Figure 4 shows the temporization done with prepared teeth.

The exocad 5XT software (Darmstadt, Germany) was used to construct ceramic laminate veneer restorations. This machine produces polychromatic, tooth-colored feldspar blanks with an integrated shade gradient, simulating the natural color play with CAD/CAM technology. The computer was used to design and mill the ceramic veneers. Following the milling process, after the initial crystallization stage, adjustment of veneers can be made at the biscuit stage of the feldspathic ceramic.

Shade, fit, marginal adaptability, shape, size, symmetry, and contacts were all evaluated. First, each was evaluated separately with glycerin acting as a holding medium. Following individual assessment, a group try-in was conducted to acknowledge the visual improvement. The patient's consent was obtained when the patient was trying in.

**Figure 5:**
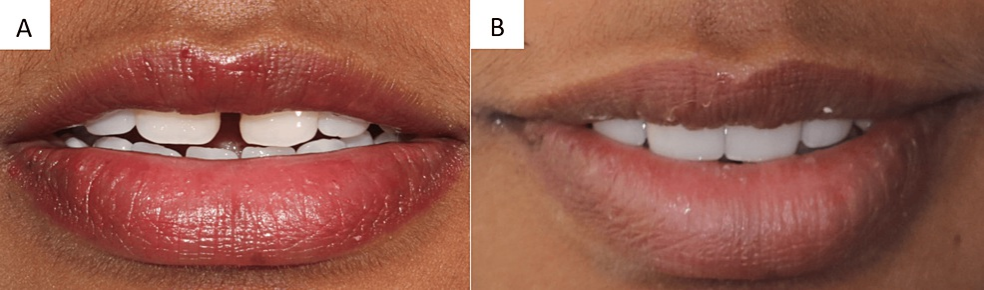
A. Preoperative photograph. B. Postoperative photograph. Image Credit: Sanket Vaidya

**Figure 6:**
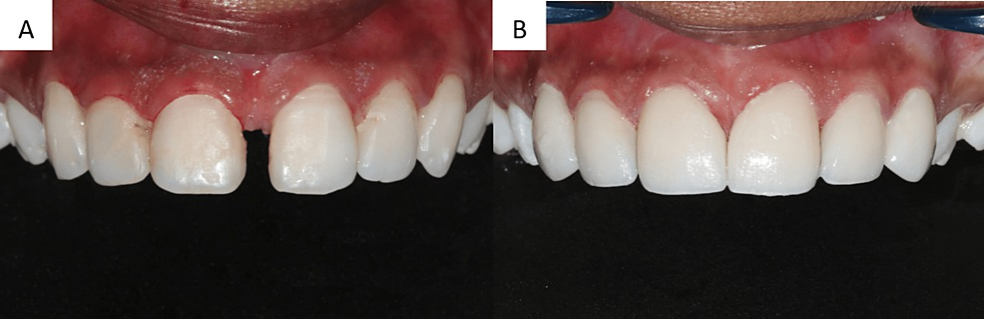
A. Smile view before the cementation of the veneer prosthesis. B. Smile view after the cementation of the veneer prosthesis. Image Credit: Sanket Vaidya

Preparing the surface involved placing the laminates on a wax sheet that represented the tooth's location in the arch to prevent mistakes and accidental breakage. For 30 seconds, 4% hydrofluoric acid was used to etch the laminates. Acid etching in dentistry is an effective way to bond different types of restorations to enamel or dentin. The "smear layer" of organic and inorganic material on a tooth, which results from cavity prep, makes for a less than ideal surface for bonding. They were properly cleaned with water after etching. The fitting surface was coated with a silane coupling agent once it had dried. First, all residue from the tooth's pre-treatment was cleaned. After that, teeth were air-dried and etched for 15 seconds using 37% phosphoric acid. Following application, 10 seconds of light curing were used to cure the polyurethane laminating adhesive bonding agent. Cementation was done using a dual-cure composite crown and resin-modified glass ionomer cement as a luting agent. After that, the veneer was carefully placed over the preparation, applying just enough pressure to let any extra cement extrude and prevent air bubbles from forming or the veneer from lifting. The laminates were first spot-cured for five seconds. After using an explorer to remove any leftover cement, the entire curing process took 20 seconds. Following luting, the patient was requested to come in for a post-treatment check-up once proper occlusion was verified. The patient was satisfied and had no complaints with the veneer prosthesis, which produced acceptable visual results as displayed in Figure 5 and Figure 6. Figure 5 represents the pre- and postoperative photographs of the patient. Figure 6 represents the smiling view before and after the cementation of the veneer prosthesis.

## Discussion

The closure of a maxillary anterior diastema has become one of the aesthetic demands of patients. Faced with these aesthetic demands, the practitioner must set up a comprehensive treatment plan that simultaneously responds to aesthetic and functional considerations. This requires good communication between the patient and the entire treatment team. Maxillary anterior diastemas can negatively affect smiles and have adverse psychological effects on an individual's social and professional life.

The following variables may be involved in the etiology of diastema: hereditary issues include congenitally missing teeth, abnormalities in the size of the teeth and jaw, additional teeth, and frenum attachments, while developmental issues include inappropriate habits, periodontal disease, tooth loss, and collapse of the posterior bite. Finding the cause of diastemas is the first step in treating them [8-10]. Prosthodontics, orthodontics, and operative dentistry are the most appropriate of the recommended treatments for diastemas [11]. One can use any of the following procedures to accomplish restorative closure of a diastema: all ceramic crowns, metal-ceramic crowns, PLVs, direct composite veneers, and composite crowns [8].

When it comes to conservative diastema closure, the materials most frequently used for veneering are ceramic and composite resin. Because of the prolonged procedure and the requirement for instant aesthetic results, adult patients may decide to avoid pursuing orthodontic treatment. The diastema was spread equally in this instance, negating the need for orthodontic treatment. Her primary demand was for the diastemas to be closed quickly with long-term aesthetic benefits [12]. The success of PLVs depends on the patient's selection; in this specific case, the patient was chosen for a conservative course of treatment due to her young age. The most appropriate course of treatment was PLVs since she had a typical overjet and overbite, a good smile line, no parafunction, and adequate enamel. Because these restorations have more chemical stability, less cytotoxicity, and a lower chance of generating irritation or sensitivity, they have the advantage of being physiologically acceptable to the body. These restorations have a smooth glazed surface that reduces plaque build-up and facilitates removal. An additional benefit is that a long-lasting repair can be achieved by combining porcelain and etched enamel with a silane coupling agent and bonding composite resin-luting agent [13-17]. Although this isn't always the case, significant large fractures can be remake, and minor pieces can be rounded off. It is best to avoid parafunction habits, like clenching or bruxism, as well as improper anatomical presentation of teeth PLVs when enamel is weak referred to as unsupported enamel rods. Veneer failure can be caused by bonding onto pre-existing composite restorations, using veneers to treat worn-down teeth with extensive dentin exposure, with poor technique. Another danger concern is the propensity of heat fluctuations to cause veneer cracking when the luting composite is thick and the porcelain is thin. When a veneer is fitted incorrectly or an excessive die spacer is used to mask underlying tooth discoloration, a thick composite layer may form. The least amount of cracking was seen when the thickness ratio of the ceramic and luting composite is not greater than 3 to 1 [18].

Composites' longevity is questionable because of their propensity for wear, minor fractures, and discoloration. Composite restorations, as opposed to PLVs, can be utilized to hide tooth discoloration or unsightly forms. The emergence of abnormally shaped teeth, such as peg laterals, midline diastema, and enamel hypoplasia, is a prevalent aesthetic concern in young people who are still conservatively treated with PLVs [19]. Porcelain laminates do have certain restrictions. When the amount of enamel is insufficient to ensure proper retention, they shouldn't be used. This isn't always the case, but veneers are probably not the best option for restoring big class 4 flaws due to the substantial volume of unsupported porcelain and the absence of tooth-colored backing. When there is a significant diastema, it is important to carefully assess the amount of unsupported porcelain. Veneers are not the best restoration for teeth with dark stains. The outlook for veneers in bruxing is questionable. After their complete restoration, these patients should undoubtedly be advised to wear a night guard [20].

A complete crown restoration is still an option for the preserved tooth if the laminates eventually fail. Veneers made of porcelain laminate provide a successful and reliable treatment option that maintains the greatest amount of healthy tooth structure. Veneers that are only partially attached to dentin have a higher chance of failing. Over 10 years, PLVs are predicted to have a 91% survival rate [21,22].

## Conclusions

The effectiveness and cosmetic value of PLVs as a beneficial treatment option in modern dentistry are further supported by this case study. Although it shows positive results, it also emphasizes how crucial thorough assessment, accurate implementation, and continuous care are to long-term success and patient happiness. Veneers made of porcelain laminate have been one of the most popular aesthetic restorations. This is among the most conservative methods of treatment. The study of aesthetics requires objectivity and effective communication between the ceramist, the patient, and dental professionals. Care must be taken when selecting cases and developing treatment plans.
